# Correlation of T1- to T2-weighted signal intensity ratio with T1- and T2-relaxation time and *IDH* mutation status in glioma

**DOI:** 10.1038/s41598-022-23527-9

**Published:** 2022-11-05

**Authors:** Takahiro Sanada, Shota Yamamoto, Mio Sakai, Toru Umehara, Hirotaka Sato, Masato Saito, Nobuyuki Mitsui, Satoru Hiroshima, Ryogo Anei, Yonehiro Kanemura, Mishie Tanino, Katsuyuki Nakanishi, Haruhiko Kishima, Manabu Kinoshita

**Affiliations:** 1 Department of Neurosurgery, Asahikawa Medical University, Midorigaoka-Higashi 2-1-1-1, Asahikawa, Hokkaido 078-8510 Japan; 2 Department of Neurosurgery, Osaka University Graduate School of Medicine, Suita, 565-0871 Japan; 3 Department of Diagnostic and Interventional Radiology, Osaka International Cancer Institute, Osaka, 541-8567 Japan; 4 Department of Neurosurgery, Hanwa Memorial Hospital, Osaka, 558-0011 Japan; 5 Department of Neurosurgery, Japanese Red Cross Kitami Hospital, Kitami, 090-8666 Japan; 6 Department of Neurosurgery, Moriyama Hospital, Asahikawa, 078-8392 Japan; 7 Department of Biomedical Research and Innovation, Institute for Clinical Research, National Hospital Organization Osaka National Hospital, Osaka, 540-0006 Japan; 8 Department of Diagnostic Pathology, Asahikawa Medical University Hospital, Asahikawa, 078-8510 Japan; 9 Department of Neurosurgery, Osaka International Cancer Institute, Osaka, 541-8567 Japan

**Keywords:** Cancer imaging, CNS cancer, Cancer imaging, CNS cancer

## Abstract

The current study aimed to test whether the ratio of T1-weighted to T2-weighted signal intensity (T1W/T2W ratio: rT1/T2) derived from conventional MRI could act as a surrogate relaxation time predictive of *IDH* mutation status in histologically lower-grade gliomas. Strong exponential correlations were found between rT1/T2 and each of T1- and T2-relaxation times in eight subjects (rT1/T2 = 1.63exp^−0.0005T1-relax^ + 0.30 and rT1/T2 = 1.27exp^−0.0081T2-relax^ + 0.48; R^2^ = 0.64 and 0.59, respectively). In a test cohort of 25 patients, mean rT1/T2 (mrT1/T2) was significantly higher in IDHwt tumors than in IDHmt tumors (*p* < 0.05) and the optimal cut-off of mrT1/T2 for discriminating IDHmt was 0.666–0.677, (AUC = 0.75, *p* < 0.05), which was validated in an external domestic cohort of 29 patients (AUC = 0.75, *p* = 0.02). However, this result was not validated in an external international cohort derived from TCIA/TCGA (AUC = 0.63, *p* = 0.08). The t-Distributed Stochastic Neighbor Embedding analysis revealed a greater diversity in image characteristics within the TCIA/TCGA cohort than in the two domestic cohorts. The failure of external validation in the TCIA/TCGA cohort could be attributed to its wider variety of original imaging characteristics.

## Introduction

Genetic characterization is an essential diagnostic work-up in patients with glioma, as presurgical prediction of the *IDH* mutation status helps choose the most appropriate treatment strategy. Rapid and aggressive surgical intervention should be pursued for IDH-wildtype (IDHwt) gliomas, which have a poor prognosis^1^. In contrast, finding an optimal balance between maximal resection and preservation of the quality of daily life is of utmost importance for IDH-mutant (IDHmt) World Health Organization (WHO) grade 2 and 3 gliomas (lower-grade gliomas: LrGGs), as these present a more favorable prognosis^1–5^. There have been great efforts within the research community to develop noninvasive imaging techniques that can predict *IDH* mutation status in gliomas, including magnetic resonance spectroscopy (MRS)^6–8^, perfusion- and diffusion-weighted magnetic resonance imaging (MRI)^9,10^, and machine learning with conventional MRI^5,11,12^. Whereas these techniques rely mainly on quantitative or semi-quantitative MRI measurement, qualitative imaging features such as the “T2 fluid-attenuated inversion recovery (FLAIR) mismatch sign” have proven to be powerful imaging surrogate markers for predicting *IDH* mutation status in tumors that appear to be histologically LrGGs^13–15^. These qualitative imaging features are derived from radiologically quantitative measurements such as the T1- and T2-relaxation time^15,16^. Ideally, tissue relaxation time should be directly measured by MR relaxometry; however, this has not yet been incorporated into routine imaging protocols as it requires additional scan time.

The ratio of T1-weighted (T1W) to T2-weighted (T2W) signal intensity (T1W/T2W ratio: rT1/T2) derived from conventional MRI has been reported as quantitative imaging technique for detecting myelin integrity in the brain^17–19^ and could potentially be used as an imaging surrogate for tissue relaxation time. The aim of the present study was to test the hypothesis that rT1/T2 is predictive of *IDH* mutation status in histologically LrGGs.

## Materials and methods

### Patient cohort

The study was approved by the Institutional Review Boards of Asahikawa Medical University Hospital (approval No. 21041) and the Osaka International Cancer Institute (No. 1612065191). The requirement for written informed consent was waived for data collected retrospectively. Patients in prospectively recruited cohorts were provided with a detailed explanation and written informed consent was obtained from each patient or their family. The investigation was performed in accordance with all relevant local guidelines and regulations, and adhered to the tenets of the Declaration of Helsinki.

**Figure 1:**
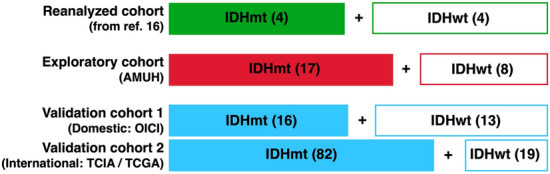
Overall study cohort. First the correlation of rT1/T2 with T1- and T2-relaxation time was investigated by reanalyzing the raw data from ref.^16^. Then, the study was conducted in two stages, as an exploratory cohort study followed by a validation cohort study to investigate the correlation of rT1/T2 and *IDH* mutation status of histologically confirmed LrGGs. IDHmt, IDH-mutant; IDHwt, IDH-wildtype; AMUH, Asahikawa Medical University Hospital; OICI, Osaka International Cancer Institute; TCIA/TCGA, Cancer Imaging Archive / Cancer Genome Atlas.

We first reanalyzed previously published T1- and T2-relaxometry data of histologically LrGGs^16^ and compared these T1- and T2-relaxometry data with rT1/T2 (the reanalyzed cohort, Fig. 1). The T1- and T2-relaxometry had been performed using MP2RAGE and multi-echo T2-weighted images of nine patients with glioma. Thus, we could access the T1- and T2-relaxometry along with the rT1/T2 data of these nine patients, and included the data of eight of these in our analyses, after excluding one patient with a K27M mutant tumor^16^.

We then prepared a new set of three cohorts and conducted a two-stage study (Fig. 1). The first cohort (exploratory cohort) comprised 25 histologically and molecularly confirmed histologically LrGGs (IDHwt: 8, IDHmt: 17) treated at Asahikawa Medical University Hospital (AMUH). The second and third cohorts were used as validation cohorts. Validation cohort 1 comprised 29 patients (IDHwt: 13, IDHmt: 16) from the Osaka International Cancer Institute (OICI) and validation cohort 2 comprised 101 patients (IDHwt: 19, IDHmt: 82) from the Cancer Imaging Archive (TCIA)/Cancer Genome Atlas (TCGA) low-grade glioma collection dataset, accessed on February 1, 2020^20,21^. Validation cohort 1 can be considered as a “domestic” and validation cohort 2 as an “international” validation cohort.

The pathological diagnosis was based on the 2016 WHO Classification of Tumors of the Central Nervous System^22^. IDHwt tumors in the present cohorts could not be fully characterized according to the 2021 WHO classification system, as molecular analyses for such as *TERT* promoter mutation, *EGFR* gene amplification, and + 7/− 10 chromosome copy-number alterations had not been performed. The inclusion criterion was availability of T1WI and T2WI. Excluded were patients with failed image co-registration or insufficient or atypical images (e.g., with tumoral hemorrhage). Table S1 provides detailed information regarding all three cohorts.

### Genetic analysis

Two laboratories performed genetic analyses of glioma tissues: the Department of Pathology, Asahikawa Medical University, Asahikawa, Japan, for the exploratory cohort; and the Department of Biomedical Research and Innovation, Institute for Clinical Research, Osaka National Hospital, Osaka, Japan, for the validation cohort 1. Immunobiological detection of *IDH1* mutation was performed for the exploratory cohort, and Sanger sequencing was performed to detect hotspot mutations of *IDH1/2* (codon 132 of *IDH1* and codon 172 of *IDH2*) for the validation cohort 1^3^. The *IDH* mutation status of tumors in the TCIA/TCGA dataset were obtained from the report by Ceccarelli et al.^23^.

### T1w/T2w (rT1/T2) image reconstruction

**Figure 2:**
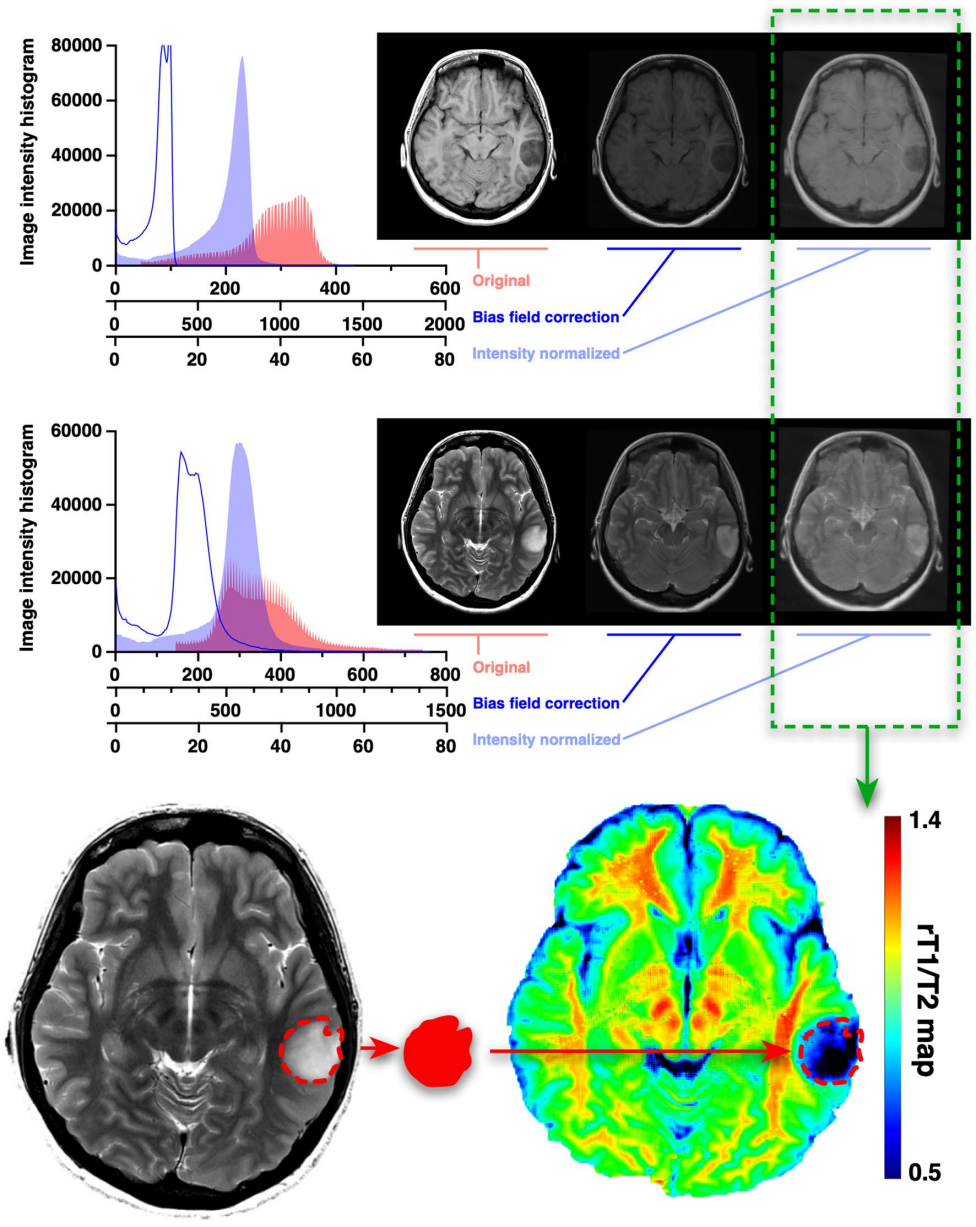
Workflow across the entire study. T1WI to T2WI signal intensity ratio (rT1/T2) images were calculated from T1- and T2-weighted images, after image normalization via bias field correction and histogram matching. Voxels-of-interest (VOIs) were defined manually based on the high-intensity area of pathological lesions on T2-weighted images, followed by mean rT1/T2 measurement within the VOI.

Table S1 lists the MRI acquisition parameters in detail. Most of the images in the exploratory cohort were acquired using General Electric 3 T scanners (Chicago, Illinois, USA), and those in the validation cohort 1 by Siemens 3 T scanners (Erlangen, Germany). Images in the TCIA cohort (the validation cohort 2) were acquired by 1.5 and 3 T scanners of various MRI vendors. T1WI and T2WI in Digital Imaging and COmmunication in Medicine (DICOM) format were converted into Neuroimaging Informatics Technology Initiative (NIfTI) format using Mango software (version 4.0.1; University of Texas Health Science Center, https://ric.uthscsa.edu/mango/mango.html, accessed on March 6, 2022). We used an in-house imaging software incorporating an algorithm for reconstructing rT1/T2 images from T1WI and T2WI^24^. The algorithm and MATLAB codes for calculating rT1/T2 can be obtained as an open-source toolbox for SPM12 developed by Ganzetti et al. (https://www.nitrc.org/projects/mrtool/, accessed on March 6, 2022)^18^. Details of the reconstruction analysis are previously reported by Ganzetti et al.^18^. Reconstruction was performed by first applying a bias field correction to the original T1WI and T2WI using SPM12 (https://www.fil.ion.ucl.ac.uk/spm/, accessed on March 6, 2022). The intensity histograms were then adjusted based on intensities extracted from non-brain tissues such as cerebrospinal fluid, bone, and soft tissues. Finally, the processed T2WI was co-registered and divided by the processed T1WI to produce an rT1/T2 image using the NIfTI “scanner-anatomical” coordinate system (Fig. 2).

### T1- and T2-relaxometry of MP2RAGE and multi-echo T2WI

Imaging was performed on a 3 T MR scanner (Prisma; Siemens Healthcare, Erlangen, Germany). T1-relaxometry was performed by converting MP2RAGE images into T1-relaxation time maps. T2-relaxometry was performed by converting multi-echo T2-weighted images into T1-relaxation time maps. In both cases, relaxometry was performed via Bayesian inference modeling (Olea Nova+; Canon Medical Systems, Tochigi, Japan). Further technical details have been reported previously^16^.

### Voxels-of-interests (VOIs) segmentation and calculation of mean rT1/T2

Author 1, with 6 years of neurosurgical experience, performed manual segmentation of the lesions by designing voxels-of-interests (VOIs) in ITK-SNAP software (version 3.8.0, http://www.itksnap.org, accessed on March 6, 2022). VOIs were designed on T2WIs with visual identification of pathologically high-intensity areas, avoiding ambiguous and vaguely abnormal lesions as much as possible (Fig. S1). The last author, with 22 years of neurosurgical experience, then evaluated the VOIs and either confirmed their position or requested modification (which occurred for five VOIs) (Table S1). The Dice similarity coefficient ranged from 0.52 to 0.81 for these VOIs (TCIA-00067, TCIA-00071, TCIA-00110, TCIA-00111, TCIA-00113). This procedure was performed on T2WI using the NIfTI “general affine transformation” coordinate system.

Each rT1/T2 image on NIfTI “scanner-anatomical” coordinate system was then co-registered with T2WI on NIfTI “general affine transformation” coordinate system using Volume Imaging in Neurological Research, Co-Registration and ROIs included (VINCI; Max Planck Institute for Neurological Research Cologne, Germany, http://www.nf.mpg.de/vinci3/, accessed on March 6, 2022), to ensure that further analysis will be performed using the NIfTI “general affine transformation” coordinate system (Fig. S2). Three-dimensional VOIs were then applied to the rT1/T2 images for calculation of mean rT1/T2 (mrT1/T2) within the VOIs (Fig. 2). VOIs were also applied to T1- and T2-relaxation time maps when those data were available.

### Image feature extraction

Image features were extracted from T1WI and T2WI according to the method described previously^25^. T1WI and T2WI were converted into 256-level grayscale images after cutoff of the upper 0.1% of signal. This procedure was not performed for the rT1/T2 images, as these are quantitative in nature. First-order texture features were calculated based on histograms of the 256-level grayscale within VOIs in the T1WI, T2WI, and rT1/T2 images. Second-order texture features were measured in Gray Level Co-occurrence Matrix (GLGM) and Gray Level Run Length Matrix (GLRLM) analyses. In total, 49 imaging features were extracted from each image (Table S2). Details of the extracted imaging features have been provided previously^25^. Texture features were used solely to compare image characteristics among the cohorts, and were not used to predict *IDH* mutation status. Such multiparametric variables would require a large training dataset to establish a reliable prediction model.

### Statistical and t-Distributed Stochastic Neighbor Embedding (t-SNE) analysis

Statistical analysis was performed using Prism 9 for macOS (GraphPad Software, San Diego, CA, USA). The relationship between mrT1/T2 and *IDH* mutation status was investigated by Mann–Whitney U test and receiver-operating characteristic (ROC) curve analysis. A *p*-value of less than 0.05 was considered significant. t-Distributed Stochastic Neighbor Embedding (t-SNE) analysis was used to investigate the difference in MRI qualities and characteristics among the three cohorts. Rtsne package version 0.15 for R with default parameters was used for this analysis (Tables S3, S4, and S5)^26^.

## Results

### Correlation between relaxometry and rT1/T2

**Figure 3:**
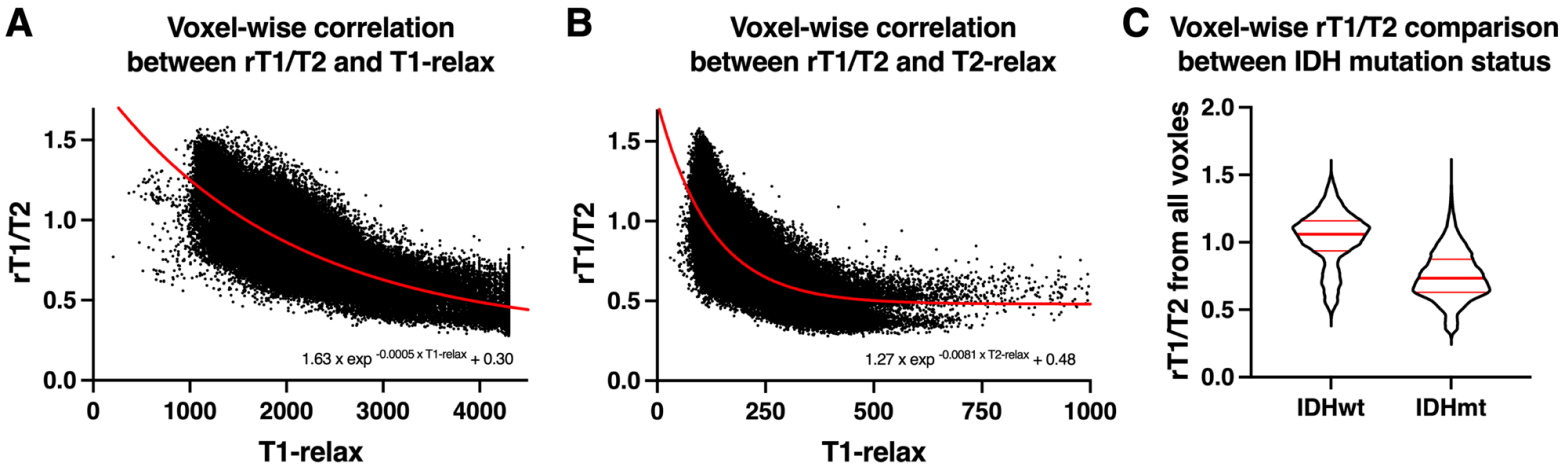
Voxel-wise comparisons between rT1/T2 and each of T1- and T2-relaxation times, according to reanalysis of data published previously (the reanalyzed cohort)^16^. The data consisted of 114,465 voxels from eight patients. T1- and T2-relaxation times both exhibited strong exponential correlations with rT1/T2 (A and B). Voxels in IDHwt tumors (26,969 voxels) showed significantly higher rT1/T2 than those in IDHmt tumors (87,496 voxels) (median value = 1.06 vs. 0.73, *p* < 0.0001) (C)^16^.

In the initial investigation of the correlation of T1- and T2-relaxation time (abbreviated as T1-relax and T2-relax, respectively) and rT1/T2 within histologically LrGG tissues, voxel-wise analysis was performed for 114,465 voxels in total. T1- and T2-relax exhibited strong exponential correlations with rT1/T2 (rT1/T2 = 1.63exp^−0.0005T1-relax^ + 0.30 and rT1/T2 = 1.27exp^−0.0081T2-relax^ + 0.48; R^2^ = 0.64 and 0.59, respectively) (Fig. 3A,B). Further comparison of the difference in rT1/T2 between IDHwt and IDHmt voxels revealed significantly higher rT1/T2 in voxels of IDHwt tumors than in those of IDHmt tumors (median value = 1.06 vs. 0.73, *p* < 0.0001) (Fig. 3C). These findings supported further investigation to test the hypothesis of rT1/T2 being an imaging surrogate for prediction of the *IDH* mutation status of histologically LrGGs.

### Determination of the cut-off value of rT1/T2 to differentiate the IDH mutation status of LrGG

**Figure 4:**
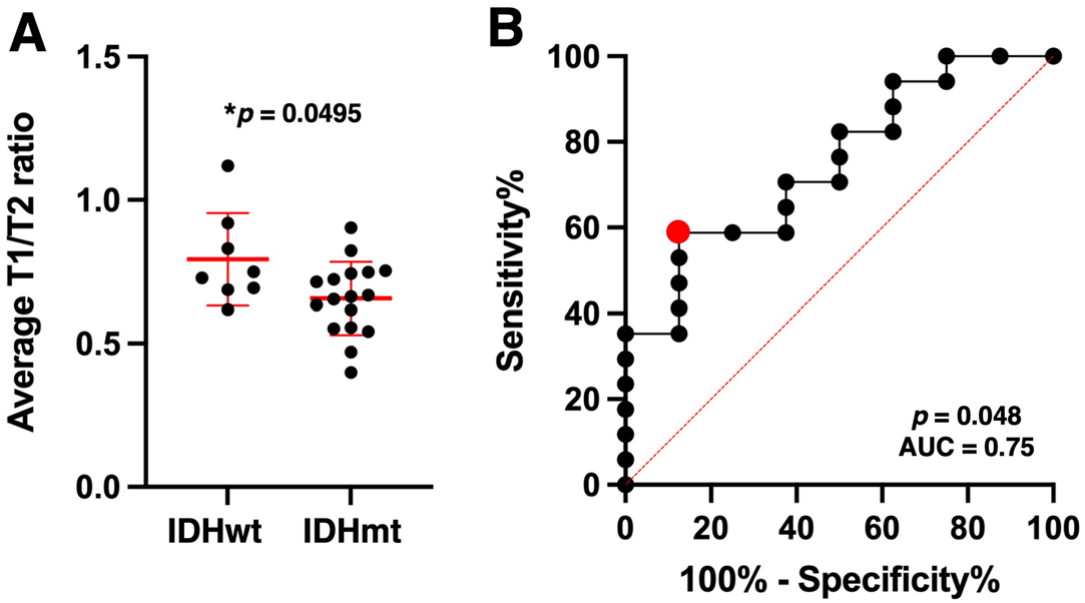
Exploration of rT1/T2 cut-off for IDH mutation in the exploratory cohort from AMUH (study 1). (**A**) There was statistically significant difference in mean rT1/T2 between the IDH-wildtype and IDH-mutant groups (*p* = 0.0495 < 0.05) (**A**). ROC curve for detecting IDH-mutant LrGGs shows AUC of 0.75, *p* = 0.0486. The red star indicates an ideal cut-off for mrT1/T2 of < 0.677, with sensitivity of 58.8% and specificity of 87.5% (**B**).

The exploratory cohort from AMUH was used to explore and determine the cut-off value of mean rT1/T2 (mrT1/T2) for differentiating IDHwt and IDHmt tumors. As shown in Fig. 4A, mrT1/T2 was significantly higher in IDHwt tumors than in IDHmt tumors (median value = 0.74 vs. 0.66, *p* < 0.05). ROC curve analysis revealed that the optimal cut-off value of mrT1/T2 for discriminating IDHmt was 0.666–0.677, with sensitivity of 58.8% and specificity of 87.5% (AUC = 0.75, *p* < 0.05) (Fig. 4B).

### Validation of the cut-off value of rT1/T2 in the domestic and TCIA/TCGA datasets

**Figure 5:**
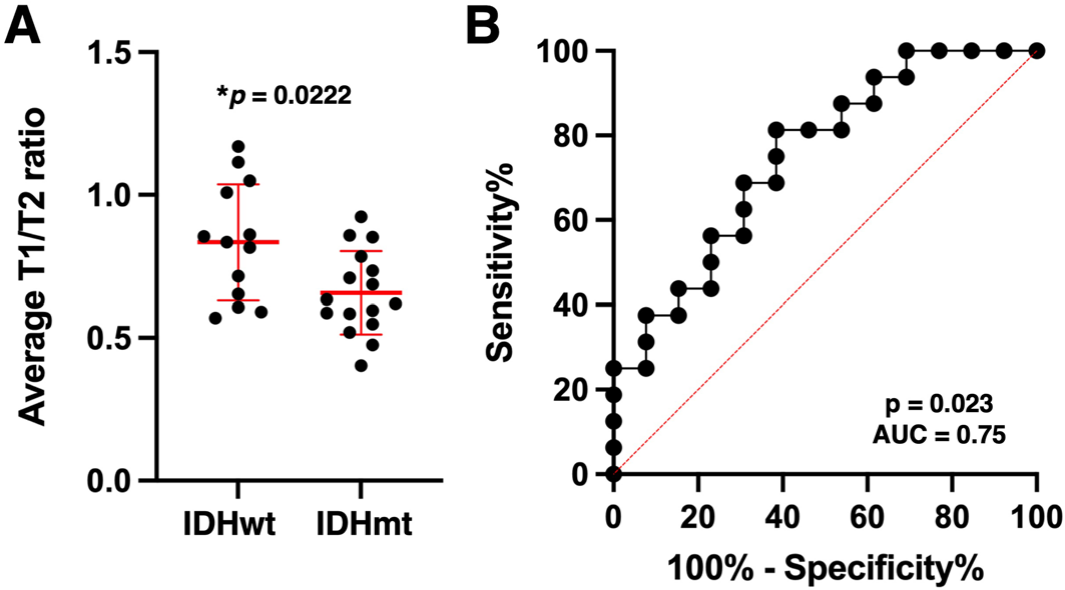
Validation of rT1/T2 cut-off for IDH mutation in the validation cohort 1 (study 2 using the domestic OICI cohort). There was statistically significant difference in mean rT1/T2 between the IDH-wildtype and IDH-mutant groups (*p* = 0.022, p < 0.05) (**A**). ROC curve for detecting IDH-mutant LrGGs shows AUC of 0.75, *p* = 0.023 (p < 0.05), with sensitivity of 56.3% and specificity of 69.2% (**B**).

The determined mrT1/T2 cut-off values were then validated using a domestic cohort (the validation cohort 1 from OICI) and an international public dataset (TCIA/TCGA cohort). The OICI cohort validated a statistically significant difference in mrT1/T2 between IDHwt and IDHmt tumors (median value = 0.83 vs. 0.63, *p* = 0.02) (Fig. 5A). ROC analysis of the OICI cohort showed AUC of 0.75 (*p* = 0.02) with sensitivity of 56.3% and specificity of 69.2%, using the previously obtained cut-off value for detecting IDHmt tumors (Fig. 5B).

**Figure 6:**
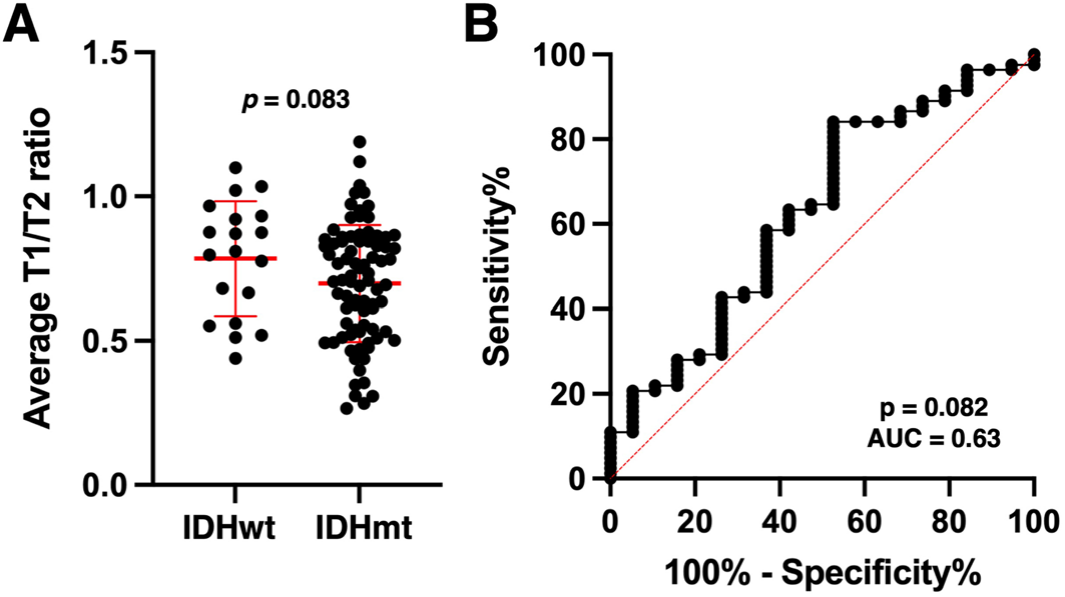
Revalidation of rT1/T2 cut-off for IDH mutation in the validation cohort 2 (study 3 using the international cohort from TICA/TCGA). There was no significant difference in mean rT1/T2 between the IDH-wildtype and IDH-mutant groups (*p* = 0.083) (**A**). ROC curve for detecting IDH-mutant LrGGs shows AUC of 0.63, (*p* = 0.082), with sensitivity of 42.9% and specificity of 73.7% (**B**).

In the validation cohort 2 (the TCIA/TCGA cohort), there was no significant difference in mrT1/T2 between IDHwt and IDHmt tumors (*p* = 0.08) (Fig. 6A). ROC analysis of the validation cohort 2 (the TCIA/TCGA cohort) showed sensitivity of 42.7% and specificity of 73.7% using the previously obtained cut-off value for detecting IDHmt LrGGs (AUC = 0.63, *p* = 0.08) (Fig. 6B).

### Impact of original image characteristics on rT1/T2 reconstruction

**Figure 7:**
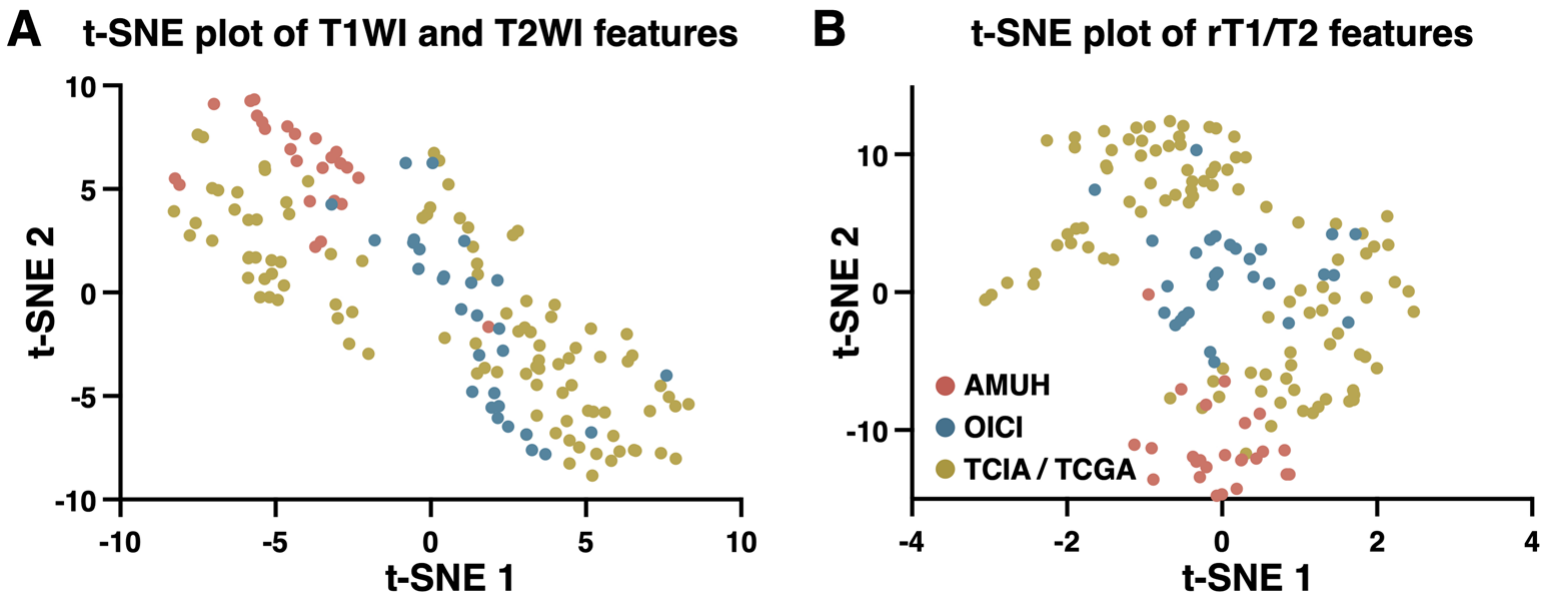
Scatter plots of the imaging texture features of T1WIs and T2WIs (**A**) and rT1/T2 (**B**) analyzed by t-SNE, and color-coded according to cohort.

We further investigated the impact of the image features in the original T1WI and T2WI for reconstructing rT1/T2 images. t-SNE analysis of the original T1WI and T2WI revealed clusters of image features in the exploratory cohort (the AMUH cohort) and the validation cohort (the OICI cohort), whereas those in the validation cohort 2 (the TCIA/TCGA cohort) were scattered across both the t-SNE1 and t-SNE axes (Fig. 7A). Reconstruction of rT1/T2 from T1WI and T2WI resulted in more dense clustering of all data points (Fig. 7B), bringing data points of the AMUH and OICI cohorts closer. However, data points obtained from the TCIA/TCGA cohort remained scattered compared to the two domestic cohorts. Further analysis revealed that the scattered data points of the TCIA/TCGA cohort were partially attributed to the variety of institutions consisting of the entire cohort (Fig. S3A). Reconstructing rT1/T2 from T1WI and T2WI was insufficient to cluster all data points, which was particularly apparent in data from Henry Ford Hospital (Fig. S3B).

t-SNE analyses of both the original T1WI and T2WI features and the rT1/T2 features of the entire cohort were unable to discriminate the *IDH* mutation status (Fig. S4).

## Discussion

Along with others, we have reported that the T1- and T2-relaxation properties of tumor tissue tightly correlate with the *IDH* mutation status of LrGGs^16,27^. These observations were obtained either by employing conventional MR relaxometry techniques^16,28^ or by newly developed magnetic resonance fingerprinting technology^29^. However, neither technology has been incorporated into routine LrGG imaging studies as yet due to additional requirements in terms of scan time^16^ or sequence and postprocessing optimization^30,31^. In contrast, conventional structural MRI (i.e., T1WI and T2WI) can partially represent the relaxation properties of a tissue following radiofrequency-induced nuclear magnetic resonance spin polarization. For example, the T2-FLAIR mismatch sign utilizes the qualitative imaging features represented on conventional structural MRI derived from quantitative tissue relaxation properties, which enable detection of IDH-mutant 1p/19q intact tumors^13–16^.

The present study aimed to explore the possibility of retrieving quantitative data from qualitative conventional structural MRI by utilizing rT1/T2, a method initially proposed as an imaging surrogate of myelin content within the brain^17^. Although the exact pathological mechanism is unknown^19,32^, the relatively high sensitivity of rT1/T2 for detecting multiple sclerosis suggests that it might be suitable for characterizing tissue microstructure within the brain^33^. As shown in Fig. 3, a good correlation was demonstrated between rT1/T2 and the T1- and T2-relaxation times of tissues. Furthermore, in the two domestic cohorts of patients with histologically LrGGs, we explored and validated that the mean value of rT1/T2 within the lesion could differentiate IDHwt from IDHmt tumors with sensitivity of 56%–59% and specificity of 69–88% (Figs. 4 and 5). These encouraging findings supported the hypothesis that rT1/T2 could be usable for the presurgical determination of *IDH* mutation status in histologically LrGGs, without performing direct measurement of the tissue relaxation properties.

However, the results obtained in the domestic cohorts (the validation cohort 1) were not reproduced in the TCIA/TCGA cohort (the validation cohort 2) (Fig. 6). Although the trend of lower rT1/T2 in IDHmt tumors than in IDHwt tumors was reproduced, the difference was much smaller compared with that found in the domestic cohorts. We consider that this discrepancy is due to differences in image quality and image characteristics between the domestic cohorts (the exploratory and validation 1 cohort) and the TCIA/TCGA cohort (the validation cohort 2). As expected, there was a greater diversity of imaging features in the TCIA/TCGA cohort than in the domestic cohorts, presumably because the TCIA/TCGA cohort contained images from four different medical institutions within the United States (Fig. 7 and Fig. S3)^34^. Table S1 shows that each institution had distinct preferences in MRI acquisition parameters, which could have impacted the final analysis. This argument is confirmed by the results of a recent multicenter clinical investigation of rT1/T2 in multiple sclerosis, which employed various MR scanners of different field strengths. A wide variation in rT1/T2 was observed throughout the entire cohort, presumably due to the heterogenic nature of the input data^35^. Thus, to use rT1/T2 for predicting *IDH* mutation status, it might be necessary to use a preset MRI acquisition protocol or an institution-dependent cut-off value.

Several limitations of the present study must be addressed. First, the sample sizes of the two domestic cohorts were relatively small, and the distributions of IDHwt and IDHmt differed among the three cohorts. This distribution difference might be responsible for the failure to validate the cut-off value in the TCIA/TCGA cohort. Ideally, a cohort with a larger sample size and an even distribution of tumors with *IDH* mutation status is necessary to validate our findings. Second, the overall sensitivity and specificity were not high enough to be acceptable in routine clinical use. The present retrospective study was unable to integrate other imaging modalities for analysis due to insufficient data; however, combining rT1/T2 with diffusion and perfusion MRI might increase diagnostic accuracy, and such research should be pursued in the future. For example, a recent study proposed the concept of combining diffusion and perfusion MRI for *IDH* mutation detection, but requires external validation^36^. Third, some of the procedures performed during rT1/T2 reconstruction are highly complicated, as mentioned by Ganzetti et al.^18^, especially image intensity normalization. The normalization method used in the present study calibrated image intensity using non-brain tissues, including cerebrospinal fluid, bone, and soft tissues; however, other methods such as deep learning could offer potential solutions to improve rT1/T2 analysis^37^. Finally, recent rapid developments in deep learning and artificial intelligence have augmented the diagnostic precision of *IDH* mutation status in glioma, with reported pooled sensitivity and specificity of 0.88 and 0.86^12^, which are much higher than those in the present study^12^. The most appropriate machine learning algorithm is yet to be determined. Therefore, the simplicity of rT1/T2 reconstructed by T1WI and T2WI alone remains appealing, withstanding the need for further improvements and validations.

## Conclusions

The findings of the present study showed that rT1/T2 strongly correlates with T1- and T2-relaxation times in histologically LrGGs. The mean value of rT1/T2 was able to discriminate IDHwt and IDHmt tumors in two domestic cohorts, with statistical significance. However, this result was not validated in the original TCIA/TCGA cohort due to the wide variety of imaging characteristics in the cohort.

## Supplementary Information

Supplementary Tables.

Supplementary Figures.

## Data Availability

The datasets used and analyzed in the current study are available from the corresponding author upon reasonable request.
