# Supplementary material for: Correlation of T1- to T2-weighted signal intensity ratio with T1- and T2-relaxation time and IDH mutation status in glioma
Source: Sci Rep. 2022 Nov 5;12:18801. doi: 10.1038/s41598-022-23527-9 (PMC9637175; doi:10.1038/s41598-022-23527-9)
Supplement: Supplementary file 2 — Supplementary Figures. [file 41598_2022_23527_MOESM2_ESM.docx]

**Correlation of T1- to T2-weighted signal intensity ratio with T1- and T2-relaxation time and *IDH* mutation status in glioma**

Takahiro Sanada^1^, Shota Yamamoto^1,2^, Mio Sakai^3^, Toru Umehara^4,2^, Hirotaka Sato^1,5^, Masato Saito^1^, Nobuyuki Mitsui^1^, Satoru Hiroshima^1^, Ryogo Anei^6^, Yonehiro Kanemura^7^, Mishie Tanino^8^, Katsuyuki Nakanishi^3^, Haruhiko Kishima^2^, Manabu Kinoshita^1,9, *^

^1^ Department of Neurosurgery, Asahikawa Medical University, Asahikawa, 078-8510, Japan

^2^ Department of Neurosurgery, Osaka University Graduate School of Medicine, Suita, 565-0871, Japan

^3^ Department of Diagnostic Radiology, Osaka International Cancer Institute, Osaka, 541-8567, Japan

^4^ Department of Neurosurgery, Hanwa Memorial Hospital, Osaka, 558-0011, Japan

^5^ Department of Neurosurgery, Japanese Red Cross Kitami Hospital, Kitami, 090-8666, Japan

^6^ Department of Neurosurgery, Moriyama Hospital, Asahikawa, 078-8392, Japan

^7^ Department of Biomedical Research and Innovation, Institute for Clinical Research, National Hospital Organization Osaka National Hospital, Osaka, 540-0006, Japan

^8^ Department of Diagnostic Pathology, Asahikawa Medical University, Asahikawa, 078-8510, Japan

^9^Department of Neurosurgery, Osaka International Cancer Institute, Osaka, 541-8567, Japan

*Corresponding author: Manabu Kinoshita M.D, Ph.D.

Department of Neurosurgery, Asahikawa Medical University

Midorigaoka-higashi 2-1-1-1, Asahikawa, Hokkaido 078-8510, Japan

Tel: +81-166-68-2594, Fax: +81-166-68-2599, Email: mail@manabukinoshita.com

**Supplementary Figure 1 (Figure S1)**

**
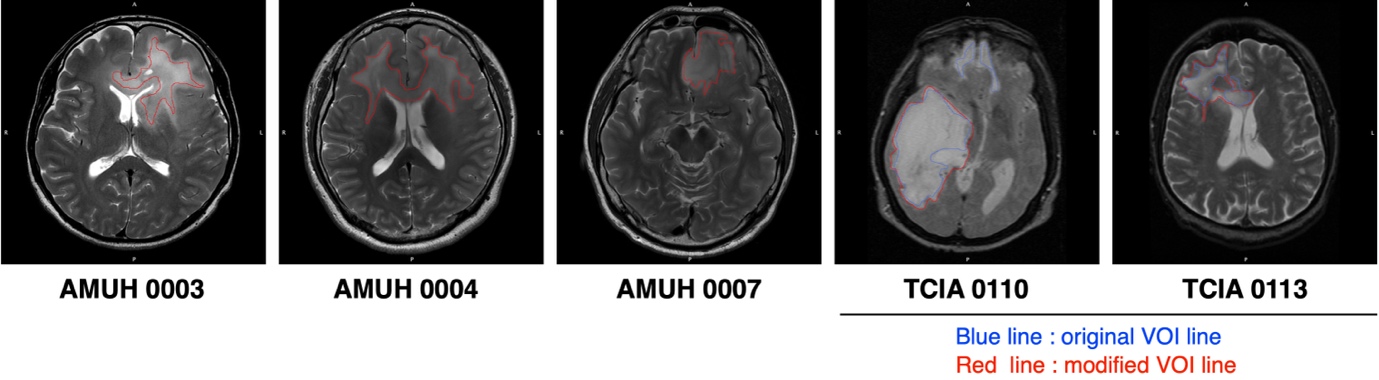
**

Examples of VOI placement including two cases in which modification of the VOI was required. The initial segmentation of pathologically high-intensity areas was performed by author 1, by careful visual inspection of T2WI, and the exclusion as much as possible of ambiguous and vague abnormal lesions. Subsequent inspection by the last author resulted in adjustment of the VOI in five cases in the TCIA cohort, including the two examples shown here.

**Supplementary Figure 2 (Figure S2)**

**
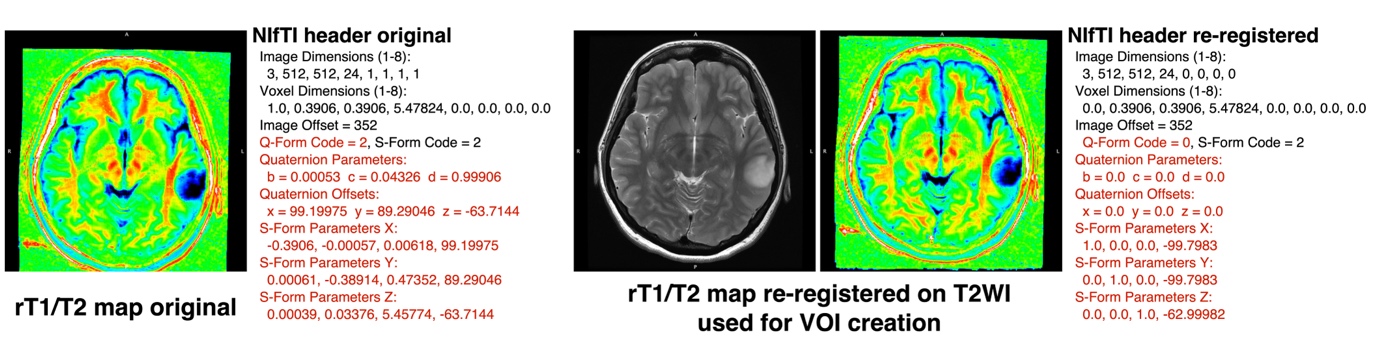
**

Re-registration of the rT1/T2 map to T2WI was necessary as the NIfTI coordinate system differed from the originally created rT1/T2 map and the T2WI used for VOI creation. The originally created rT1/T2 map used the “scanner-anatomical” coordinates using qform, while the T2WI for VOI creation used the “general affine transformation” coordinates using sform.

**Supplementary Figure 3 (Figure S3)**

**
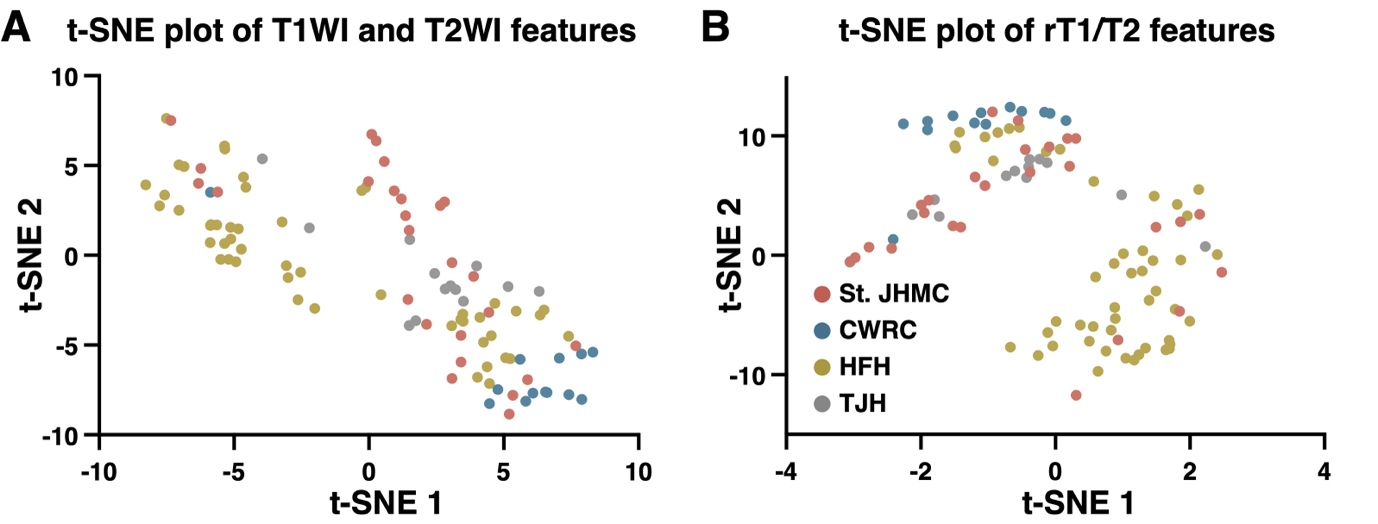
**

Scatter plots of the imaging texture features of T1WIs and T2WIs (A) and rT1/T2 (B) analyzed by t-SNE, and color-coded according to institution. St. Joseph Hospital/Medical Center, St. JHMC; Case Western Reserve University, CWRU; Henry Ford Hospital, HFH; Thomas Jefferson University, TJU.

**Supplementary Figure 4 (Figure S4)**

**
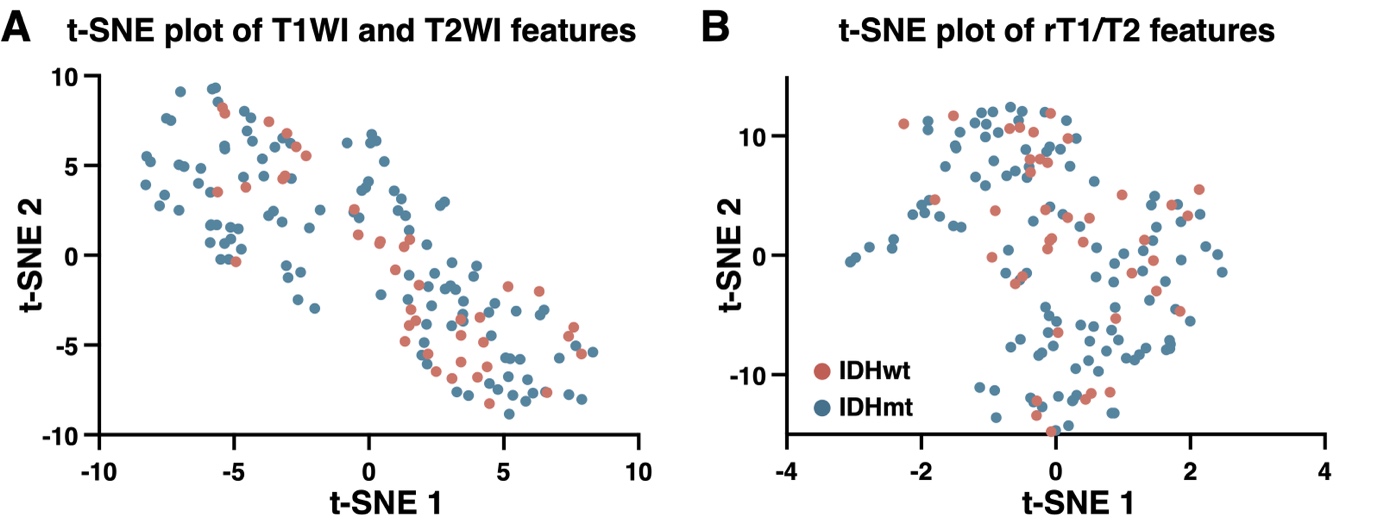
**

Scatter plots of the imaging texture features of T1WIs and T2WIs (A) and rT1/T2 (B) analyzed by t-SNE, and color-coded according to tumor *IDH* mutation status.
